# Effects of exercise training on ANGPTL3/8 and ANGPTL4/8 and their associations with cardiometabolic traits

**DOI:** 10.1016/j.jlr.2023.100495

**Published:** 2023-12-29

**Authors:** William G. Hoffmann, Yan Q. Chen, Charles S. Schwartz, Jacob L. Barber, Prasun K. Dev, Riley J. Reasons, Juan S. Miranda Maravi, Bridget Armstrong, Robert E. Gerszten, Günther Silbernagel, Robert J. Konrad, Claude Bouchard, Mark A. Sarzynski

**Affiliations:** 1 University of South Carolina School of Medicine, Columbia, SC, USA; 2 Lilly Research Laboratories, Eli Lilly and Company, Indianapolis, IN, USA; 3 Department of Exercise Science, University of South Carolina, Columbia, SC, USA; 4 Division of Cardiovascular Medicine, Beth Israel Deaconess Medical Center, Boston, MA, USA; 5 Division of Vascular Medicine, Department of Internal Medicine, Medical University of Graz, Graz, Austria; 6 Human Genomics Laboratory, Pennington Biomedical Research Center, Baton Rouge, LA, USA

**Keywords:** lipase/lipoprotein, lipolysis and fatty acid metabolism, lipids, triglycerides, lipoproteins/metabolism, exercise intervention, LPL, angiopoietin-like proteins, lipid metabolism

## Abstract

Angiopoietin-like protein (ANGPTL) complexes 3/8 and 4/8 are established inhibitors of LPL and novel therapeutic targets for dyslipidemia. However, the effects of regular exercise on ANGPTL3/8 and ANGPTL4/8 are unknown. We characterized ANGPTL3/8 and ANGPTL4/8 and their relationship with in vivo measurements of lipase activities and cardiometabolic traits before and after a 5-month endurance exercise training intervention in 642 adults from the HERITAGE (HEalth, RIsk factors, exercise Training And GEnetics) Family Study. At baseline, higher levels of both ANGPTL3/8 and ANGPTL4/8 were associated with a worse lipid, lipoprotein, and cardiometabolic profile, with only ANGPTL3/8 associated with postheparin LPL and HL activities. ANGPTL3/8 significantly decreased with exercise training, which corresponded with increases in LPL activity and decreases in HL activity, plasma triglycerides, apoB, visceral fat, and fasting insulin (all *P* < 5.1 × 10^−4^). Exercise-induced changes in ANGPTL4/8 were directly correlated to concomitant changes in total cholesterol, LDL-C, apoB, and HDL-triglycerides and inversely related to change in insulin sensitivity index (all *P* < 7.0 × 10^−4^). In conclusion, exercise-induced decreases in ANGPTL3/8 and ANGPTL4/8 were related to concomitant improvements in lipase activity, lipid profile, and cardiometabolic risk factors. These findings reveal the ANGPTL3-4-8 model as a potential molecular mechanism contributing to adaptations in lipid metabolism in response to exercise training.

Angiopoietin-like proteins (ANGPTLs) are a family of proteins structurally similar to angiopoietins. Among them, ANGPTL3, ANGPTL4, and ANGPTL8 are known inhibitors of LPL activity and therefore increase circulating triglyceride (TG) levels (1, 2, 3, 4, 5, 6).

ANGPTL4 is expressed in numerous tissues, including liver, adipose tissue, kidney, pancreas, and skeletal muscle, and inactivates LPL under conditions of fasting and exercise by binding to the catalytic domain of LPL and unfolding the active portion of the protein (7, 8, 9). ANGPTL4 is upregulated in resting muscles during exercise, which reduces LPL activity and thus helps direct TG-derived fatty acids to active skeletal muscle as fuel. Conversely, ANGPTL3 is primarily expressed in the liver and reduces LPL activity in oxidative tissues in the fed state, thereby directing fatty acids to adipose tissue for storage (10). Both ANGPTL3 and ANGPTL4 have been utilized as novel therapeutic targets for the treatment of dyslipidemia (11, 12). ANGPTL8 is mostly expressed in the liver and adipose tissue and was originally described as an atypical ANGPTL protein, as it lacks the fibrinogen-like C-terminal domain found in other ANGPTL members (2). Importantly, ANGPTL8 acts as a nutrient sensor to form complexes with ANGPTL3 and ANGPTL4 to increase and decrease, respectively, their LPL inhibitory activities to partition fatty acids to adipose tissue or skeletal muscle under feeding or fasting conditions, respectively (13, 14, 15, 16, 17). When complexed with ANGPTL8, LPL inhibition by ANGPTL4 is repressed, whereas LPL inhibition by ANGPTL3 is enhanced (13, 15, 18, 19).

Few studies have characterized ANGPTL complexes in human serum. Chen *et al.* (15) measured the complexes in 352 healthy adults and found that the levels of ANGPTL3/8 and ANGPTL4/8 were related to markers of the metabolic syndrome. A study of 93 patients with type 2 diabetes and 99 healthy adult controls found that ANGPTL3/8 levels were similar between type 2 diabetes patients and controls, whereas ANGPTL4/8 levels were about twofold higher in patients (20). The authors found correlations of both complexes with multiple lipid markers in type 2 diabetes patients to be similar to their previous study (15) of healthy adults. To our knowledge, no study has examined the relationship between ANGPTL3/8 and ANGPTL4/8 with lipase activities, complex measures of lipid and lipoprotein subclasses, and/or data from glucose tolerance tests in humans.

Given the importance of ANGPTL3/8 and ANGPTL4/8 complexes on regulation of LPL activity, they represent novel therapeutic targets. Recently, an anti-ANGPTL3/8 antibody was shown to block ANGPTL3/8-mediated LPL inhibition in vitro and potently lower TG in vivo (21). Moreover, a new phase I trial showed that a single dose of a monoclonal antibody against the ANGPTL3/8 complex reduced TG and TG-rich lipoproteins (TRLPs) in patients with hyperlipidemia (22). On the other hand, lifestyle modification, including regular exercise, is considered a first-line therapy for elevated serum TG (23), as exercise is well known to cause long-lasting reductions in fasting and postprandial TG levels (24, 25). Exercise is also known to increase LPL activity (26, 27, 28) and activate ANGPTL4 (8, 29, 30). However, the mechanisms by which exercise lowers TG, including increasing LPL activity, have not been fully elucidated and may involve ANGPTL3/8 and ANGPTL4/8. To date, no study has examined the effects of regular exercise on ANGPTL3/8 and ANGPTL4/8 concentrations.

Thus, the current study sought to characterize ANGPTL3/8 and ANGPTL4/8 complexes before and after exercise training in a large study of healthy adults and relate these measures to concomitant in vivo measurements of postheparin (PH) lipase activities as well as thorough lipid, lipoprotein, body composition, and insulin and glucose profiling.

## Materials and methods

### HERITAGE family study

#### Study design

The HEalth, RIsk factors, exercise Training And GEnetics (HERITAGE) Family Study recruited 855 self-identified black and white adults from 218 family units to one of four clinical centers (Laval University, Quebec; University of Minnesota; University of Texas; and Arizona State University and then Indiana University) to complete a 20-week endurance exercise training program during 1993–1997 (Clinical trial registration number: #NCT00005137). This was a single-arm intervention (i.e., nonrandomized) with no control group. The CERT (Consensus on Exercise Reporting Template) (31) guidelines for this study are reported in supplemental Table S1. Full details on study design and sample have been previously described (32, 33). Briefly, inclusion and exclusion criteria included age (17–65 years), physical activity level (physically inactive the previous 3 months), BMI below 40 kg/m^2^, normotensive or mildly hypertensive (<160/100 mm Hg), not taking medications for hypertension, diabetes, or dyslipidemia, and no history of certain medical conditions. The study protocol had been approved by the Institutional Review Boards at each of the participating centers of the HERITAGE Family Study consortium. Written informed consent was obtained from each participant. All research was performed in accordance with the Declaration of Helsinki.

#### Exercise training program

All included participants completed 20 weeks of a supervised, progressive, endurance training program using the same standardized protocol at the four clinical centers. Participants exercised three times per week on cycle ergometers (Universal Aerobicycles, Cedar Rapids, IA) at an exercise intensity based on their maximal oxygen uptake (VO_2_max) at baseline. Participants exercised at the heart rate associated with 55% of their baseline VO_2_max for 30 min per session for the first 2 weeks, and the duration and intensity were gradually increased every 2 weeks until reaching 75% VO_2_max for 50 min per session for the final 6 weeks of training.

#### Final sample size for current study

A total of 742 adults from 204 family units were considered completers, as they finished the training program (completed at least 95% or 57 of the 60 required training sessions). A summary of reasons for not completing the study and/or dropping out are described in supplemental Table S1. From these 742 completers, the current analysis utilized data from 642 participants with complete data on ANGPTL3/8 and ANGPTL4/8 and lipids before and after training.

#### Measurement of ANGPTL3/8 and ANGPTL4/8 complexes

ANGPTL3/8 and ANGPTL4/8 complexes were measured with dedicated MesoScale Discovery electrochemiluminescence immunoassays as previously described (15). For the ANGPTL3/8 assay, the capture antibody recognized ANGPTL8, and the detection antibody recognized ANGPTL3. For the ANGPTL4/8 assay, the capture antibody recognized ANGPTL4, and the detection antibody recognized ANGPTL8. Both assays are reproducible, with interassay coefficients of variation less than 10%.

#### Determination of plasma lipids, lipoproteins, and PH lipolytic activities

Plasma samples were taken in the morning following a 12 h fast twice at baseline and 24 h and 72 h after the last exercise session. For eumenorrheic women, to ensure samples were collected at the same phase of the menstrual cycle, all samples were obtained in the early follicular phase. Whole blood samples were ultracentrifuged to isolate VLDL. The HDL fraction was obtained after precipitation of LDL in the infranatant by the heparin manganese chloride method (34). The HDL_2_ and HDL_3_ subfractions were selectively precipitated from the infranatant using dextran sulfate. Total cholesterol and TG levels were determined in plasma and lipoprotein fractions by enzymatic methods using the Technicon RA-1000 analyzer. Concentrations of apoA-I and apoB in plasma and lipoprotein fractions were measured by the rocket-immunoelectrophoretic method. For each time point (baseline and post-training), the two values were averaged and used for analyses. Lipoprotein traits were adjusted for changes in exercise-induced changes in hemodilution.

PH LPL and HL activities were measured on one occasion before and after completion of the exercise program, separate from the blood draw for lipid measures, after a 12 h overnight fast, 10 min after an intravenous injection of heparin (60 IU/kg body mass). The PH-lipolytic activities were measured using a modification of the method of Nilsson-Ehle and Ekman, as previously described (35). Extensive quality-control procedures were implemented to ensure high quality and reproducible lipid and lipase assays (36, 37).

The lipoprotein subclass profile was quantified via NMR spectroscopy at LabCorp, Inc (Morrisville, NC) using the LP4 deconvolution algorithm (38).

#### Body composition measures

Body composition measurements were collected once at baseline and post-training, as previously described (39). In addition to anthropometric and girth measurements, body density, fat-free mass, fat mass, and percent body fat were assessed through hydrostatic weighing. Computed axial tomography scans were performed to calculate abdominal visceral, subcutaneous, and total fat areas as previously described (40).

#### Intravenous glucose tolerance test (IVGTT) protocol

Fasting plasma glucose and insulin were assayed in the fasted state at baseline and 24–36 h after the last exercise training session. An intravenous glucose tolerance test (IVGTT) was performed before and 24 h post-training with blood samples collected at 16 time points over 3 h. Insulin sensitivity index, acute insulin response to glucose (AIR_g_), glucose effectiveness, and the disposition index were derived using the MINMOD software (version 5.18; MINMOD Millennium) (41). AIR_g_ was calculated as the area under the insulin curve for the first 10 min and when multiplied with insulin sensitivity index produced the estimated disposition index as an indicator of the ability of the pancreatic beta cells to compensate for changes in insulin sensitivity.

For all variables, the change with training (delta) was calculated by subtracting the baseline value from the post-training value. Detailed protocols, quality control, reproducibility of measures, and training response of the included cardiometabolic variables have previously been summarized (33).

### Statistical analysis

The present study represents analysis of existing and new (ANGPTL complex) data from the HERITAGE exercise intervention. Two-sample *t*-tests were used to compare ANGPTL3/8 and ANGPTL4/8 levels between sex and self-reported race groups. Analysis of variance models were used to compare ANGPTL complex levels between four combined race-sex groups. Paired *t*-tests were used to examine changes in ANGPTL3/8 and ANGPTL4/8 with exercise training. Pearson correlations were used to examine the relationships of the ANGPTL complexes with each other and with clinical phenotypes at baseline and in response to training (i.e., change in ANGPTL complex with change in trait). Linear mixed models (proc mixed in SAS) were performed to examine mean differences in clinical phenotypes across quintiles of ANGPTL3/8 and ANGPTL4/8. Nonindependence among family members was adjusted for by using a “sandwich estimator,” which asymptotically yields the same parameter estimates as ordinary least squares or regression methods, but the standard errors and consequently hypothesis tests are adjusted for the dependencies. The method is general, assuming the same degree of dependency among all members within a family. All baseline analyses (correlations and mixed models) included age, sex, and race as covariates. Analyses for change phenotypes also included baseline trait value as a covariate to represent the true association of change in phenotype with change in ANGPTL independent of their starting values. A total of 54 clinical traits were examined across the categories of lipids, lipoproteins, body composition, and insulin/glucose. As such, we used a Bonferroni-adjusted *P* value threshold of <9.3 × 10^−04^ to signify statistical significance for the individual trait correlation and general linear model analyses.

Exploratory regression models were performed to identify change phenotypes that predict the changes in ANGPTL complexes. For each ANGPTL complex trait, any phenotype showing a nominal (*P* < 0.05) correlation with it was entered into a multivariable forward regression model along with age, sex, and race. Regression models for change phenotypes that predict change in ANGPTL complexes also included the respective baseline ANGPTL complex value. All analyses were performed using SAS 9.4 (Cary, NC).

## Results

### ANGPTL3/8 and ANGPTL4/8 levels—baseline

Mean values for ANGPTL3/8 and ANGPTL4/8 before and after exercise training for the total sample and within sex and race subgroups are shown in Table 1. At baseline, the mean (standard deviation) concentrations of ANGPTL3/8 and ANGPTL4/8 were 13.3 (7.2) and 13.6 (8.0) ng/ml, respectively. At baseline, ANGPTL4/8 differed across race groups, with black participants having higher values than white participants. At baseline, white female participants had significantly lower mean ANGPTL4/8 values compared with all other race-sex subgroups (Table 1).

**Table 1:** Mean values of ANGPTL3/8 and ANGPTL4/8 at baseline and post-training in the total HERITAGE sample and by sex and race subgroups

	ANGPTL3/8, ng/ml			ANGPTL4/8, ng/ml		
Group	Baseline	Post	Delta	Baseline	Post	Delta
Total (N = 642)	13.3 (7.2)	12.5 (7.2)	−0.8 (6.4)^a^	13.6 (8.0)	13.1 (8.2)	−0.5 (8.5)
Females (*n* = 358)	13.3 (6.8)	12.5 (6.9)	−0.7 (6.3)^a^	13.1 (8.5)	13.4 (9.1)	0.2 (9.5)
Males (*n* = 284)	13.3 (7.6)	12.4 (7.6)	−0.8 (6.7)^a^	14.1 (7.5)	12.8 (6.9)	−1.3 (7.1)^a^^,^^c^
Blacks (*n* = 228)	13.5 (7.2)	13.1 (7)	−0.3 (6.9)	14.6 (9.0)^b^	13.7 (8.2)	−0.9 (9.2)
Whites (*n* = 414)	13.2 (7.2)	12.1 (7.3)	−1.0 (6.2)^a^	13.0 (7.4)	12.8 (8.2)	−0.2 (8.1)
Black females (*n* = 146)	13.6 (6.9)	13.5 (7.1)	−0.1 (6.9)	14.5 (9.4)	14.2 (9.5)	−0.3 (10.1)
Black males (*n* = 82)	13.2 (7.7)	12.4 (6.8)	−0.8 (7)	14.8 (8.5)	12.8 (5.4)	−2.0 (7.3)^a^
White females (*n* = 212)	13.1 (6.8)	11.9 (6.7)	−1.2 (5.7)^a^	12.2 (7.6)^d^	12.8 (8.8)	0.6 (9)
White males (*n* = 202)	13.3 (7.6)	12.4 (8)	−0.9 (6.6)	13.8 (7)	12.8 (7.5)	−1.1 (7)^a^

Delta = baseline value subtracted from post-training value.

a *P* < 0.05 for within-group change with exercise training.

b *P* = 0.02 for mean difference compared with white participants.

c *P* = 0.02 for mean difference compared with female participants.

d *P* = 0.02 for mean difference compared with all other race-sex subgroups.

The two complexes showed a moderate correlation with each other at baseline (*r* = 0.28, *P* = 3.4 × 10^−10^). There were no differences in the distribution of race or sex across quintiles of ANGPTL3/8 and ANGPTL4/8 (Tables 2 and 3), although the proportion of black participants in ANGPTL4/8 quintile 5 (Q5; 42%) was significantly higher than in Q1 (27%; *P* = 0.009).

**Table 2:** Cardiometabolic profile of HERITAGE subjects at baseline and post-training according to baseline quintiles of the ANGPTL3/8 complex

ANGPTL3/8 baseline quintile	1 (*n* = 128)		2 (*n* = 129)		3 (*n* = 128)		4 (*n* = 129)		5 (*n* = 128)		Group differences^b^			Correlation^c^			
														Baseline^d^		Post-training^e^	
	Baseline	Post	Baseline	Post	Baseline	Post	Baseline	Post	Baseline	Post	Baseline^d^	Post^d^	Post^e^	*r*	*P*	*r*	*P*
ANGPTL3/8 range, ng/ml	1.27–7.46		7.48–10.26		10.27–13.39		13.39–18.04		18.07–45.55								
ANGPTL3/8, ng/ml	5.41 (1.43)	7.59 (3.75)^a^	8.87 (0.79)	9.95 (5.1)^a^	12.01 (0.9)	11.47 (4.75)	15.59 (1.3)	14.04 (5.69)^a^	24.51 (6.29)	19.39 (9.22)^a^	6.5E-145	1.5E-30	0.96				
ANGPTL4/8, ng/ml	11.41 (7.88)	10.8 (7.03)	12.19 (6.69)	12.65 (7.6)	13.1 (8.76)	12.3 (6.33)	13.28 (6.42)	13.89 (8.45)	17.94 (8.61)	15.95 (10.22)	1.04E-06	1.1E-04	0.03	0.28	3.4E-10	0.25	1.8E-10
Female (%)	61 (48)		81 (63)		70 (55)		79 (61)		67 (52)		0.08						
Black (%)	44 (34)		42 (33)		45 (35)		48 (37)		49 (38)		0.88						
Age, years	30.1 (11.8)	30.1 (11.8)	33.8 (13.5)	33.8 (13.5)	35.6 (13.4)	35.6 (13.4)	36.3 (13.7)	36.3 (13.7)	38.5 (13.4)	38.5 (13.4)	0.29			0.21	1.6E-07	0.11	0.005
BMI, kg/m^2^	23.7 (3.8)	23.4 (3.7)^a^	25.1 (4.6)	24.9 (4.4)	26.4 (4.7)	26.3 (4.5)	27.7 (5.9)	27.7 (5.7)	29.3 (5.7)	29.2 (5.6)	5.67E-12	2.2E-12	0.08	0.32	4.4E-13	0.12	0.003
Visceral fat, cm^2^	56.8 (35.7)	52.4 (31.7)	71 (47)	65.3 (43.6)	81.4 (47.4)	76.3 (47.1)	93.1 (54.6)	88.3 (51.2)	116.3 (69.9)	108.5 (64.3)	3.2E-12	2.9E-14	0.21	0.34	5.0E-15	0.143	3.8E-04
Total TG, mmol/l	0.93 (0.40)	0.93 (0.49)	1.03 (0.51)	1.08 (0.60)	1.22 (0.61)	1.15 (0.58)^a^	1.38 (0.72)	1.33 (0.70)	1.78 (1.08)	1.68 (1.12)^a^	2.96E-17	5.6E-12	0.25	0.39	4.0E-19	0.14	4.4E-04
PH-LPL activity, nm/ml/min	69.2 (36)	73.6 (29.5)	63.5 (32.5)	70.8 (35.5)^a^	57.3 (29.5)	65.8 (27.2)^a^	55.7 (31.1)	66.9 (28.6)^a^	56.2 (27.2)	63.5 (25)^a^	1.92E-04	0.01	0.80	−0.23	2.2E-07	−0.13	0.002
PH-HL activity, nm/ml/min	179 (70.2)	169.4 (66.6)^a^	186.0 (72.4)	173.8 (72.1)^a^	187.4 (73.4)	174.9 (71.7)^a^	192.4 (74.4)	179.4 (68.7)^a^	199 (73.5)	191.7 (73)	0.004	0.03	0.24	0.22	1.1E-06	0.15	2.1E-04
Fasting insulin, pmol/l	54.44 (31.8)	49.9 (24.4)	56.54 (21.8)	54.51 (23.9)	68.39 (52.9)	59.21 (34.6)^a^	85.42 (54.4)	74.21 (48.3)^a^	85.59 (60.1)	75.8 (42.8)^a^	6.0E-10	1.2E-07	0.07	0.24	8.9E-08	0.21	4.7E-07
Insulin sensitivity index (Si), 10^−4^ min^−1^·(μU/ml)^−1^	4.75 (3.0)	5.0 (3.0)	4.43 (2.8)	4.98 (2.9)	3.71 (2.6)	4.33 (2.8)^a^	3.58 (3.1)	3.34 (2.0)	2.84 (2.3)	3.19 (2.3)^a^	7.3E-06	2.2E-07	3.4E-05	−0.21	4.2E-06	−0.12	0.007

Values listed as mean (SD) for continuous variables and *N* (%) for categorical.

a *P* < 0.05 for within-group change with exercise training.

b *P* for differences across the five groups calculated with Chi-square test and ANCOVA for categorical and continuous variables, respectively.

c Pearson's *r* value for correlation between ANGPTL3/8 and clinical phenotypes as continuous variables.

d Mixed models adjusted for age, sex, race, and family membership.

e Primary post-training models also adjusted for baseline trait value (to represent association of change with change).

**Table 3:** Cardiometabolic profile of HERITAGE subjects at baseline and post-training according to baseline quintiles of the ANGPTL4/8 complex

ANGPTL4/8 baseline quintile	1 (*n* = 128)		2 (*n* = 129)		3 (*n* = 128)		4 (*n* = 129)		5 (*n* = 128)		Group differences^b^			Correlation^c^			
														Baseline^d^		Post-training^e^	
	Baseline	Post	Baseline	Post	Baseline	Post	Baseline	Post	Baseline	Post	Baseline^d^	Post^d^	Post^e^	*r*	*P*	*r*	*P*
ANGPTL4/8 range, ng/ml	0.74–7.33		7.36–10.74		10.77–13.37		13.38–18.55		18.57–73.78								
ANGPTL4/8, ng/ml	5.19 (1.54)	7.91 (5.1)^a^	9.00 (0.90)	11.58 (8.64)^a^	12.04 (0.77)	12.7 (6.58)	15.65 (1.49)	14.13 (5.93)^a^	26.05 (8.01)	19.29 (9.46)^a^	1.2E-128	5.1E-30	0.004				
ANGPTL3/8, ng/ml	10.25 (5.18)	10.4 (6.88)	12.4 (6.52)	11.77 (5.78)	12.38 (5.42)	11.52 (4.85)	14.76 (7.81)	12.96 (7.86)	16.59 (8.75)	15.78 (8.88)	2.4E-08	5.1E-04	0.11	0.28	3.4E-10	0.25	1.8E-10
Female (%)	84 (66)		87 (67)		83 (65)		81 (63)		79 (62)								
Black (%)	34 (27)		48 (37)		45 (35)		47 (36)		54 (42)								
Age, years	35.8 (13.2)	35.8 (13.2)	35.0 (13.4)	35.0 (13.4)	31.4 (11.6)	31.4 (11.6)	35.3 (14.3)	35.3 (14.3)	36.8 (14.1)	36.8 (14.1)	0.04			0.02	0.53	−0.04	0.37
BMI, kg/m^2^	24.9 (4.8)	24.8 (4.7)	25.5 (4.9)	25.4 (4.8)	26.1 (4.7)	25.9 (4.5)	26.8 (5.4)	26.6 (5.4)^a^	29.0 (6.0)	28.8 (5.9)	6.14E-05	4.9E-05	0.83	0.20	1.2E-05	0.10	0.02
Visceral fat, cm^2^	71.8 (42.5)	67.8 (41.4)^a^	77.1 (51.7)	72.5 (49.8)^a^	78.5 (48.9)	73.1 (46.0)^a^	86.9 (55.6)	80.6 (52.8)^a^	103.9 (71.1)	96.2 (64.0)^a^	8.6E-05	1.3E-04	0.99	0.20	1.1E-05	0.06	0.16
Total TG, mmol/l	1.13 (0.58)	1.14 (0.62)	1.14 (0.63)	1.07 (0.57)^a^	1.18 (0.57)	1.15 (0.56)	1.25 (0.72)	1.23 (0.71)	1.63 (1.09)	1.59 (1.15)	3.0E-04	3.5E-04	0.24	0.21	2.1E-06	0.07	0.08
PH-LPL activity, nm/ml/min	64 (37.8)	72.8 (32.4)^a^	61.6 (31.3)	71.6 (30.8)^a^	55.9 (28.2)	62.9 (27.8)^a^	57.9 (29.3)	67.7 (26.9)^a^	62.6 (31.3)	66.1 (29)	0.41	0.13	0.29	−0.06	0.20	0.04	0.33
PH-HL activity, nm/ml/min	179.6 (68.4)	169.9 (66.9)	184.6 (74.3)	172.7 (67.7)^a^	201.0 (72.6)	189.0 (73.9)^a^	193.6 (70.7)	178.5 (67.2)^a^	184.9 (77.1)	178.6 (76.6)	0.50	0.81	0.44	0.05	0.30	−0.11	0.01
Fasting insulin, pmol/l	56.18 (23.6)	48.92 (21.8)^a^	59.33 (37.1)	57.3 (26.3)	68.56 (40.1)	61.57 (35.8)	74.67 (43.8)	67.61 (42.5)^a^	91.94 (75.0)	80.21 (49.5)	2.57E-05	7.0E-07	0.006	0.27	8.7E-10	0.11	0.009
Insulin sensitivity index (Si), 10^−4^ min^−1^·(μU/ml)^−1^	5.16 (3.4)	5.01 (3.0)	4.36 (3.1)	4.63 (3.0)	3.58 (2.3)	3.99 (2.3)	3.43 (2.4)	3.79 (2.6)	2.75 (2.2)	3.33 (2.4)^a^	5.1E-08	4.3E-04	0.48	−0.21	3.0E-06	−0.17	5.7E-05

Values listed as mean (SD) for continuous variables and *N* (%) for categorical.

a *P* < 0.05 for within-group change with exercise training.

b *P* for differences across the five groups calculated with Chi-square test and ANCOVA for categorical and continuous variables, respectively.

c Pearson's *r* value for correlation between ANGPTL4/8 and clinical phenotypes as continuous variables.

d Mixed models adjusted for age, sex, race, and family membership.

e Primary post-training models also adjusted for baseline trait value (to represent association of change with change).

### Associations with cardiometabolic traits—baseline

Baseline cardiometabolic profiles of the 642 participants according to baseline quintiles of ANGPTL3/8 and ANGPTL4/8 can be found in Tables 2 and 3, respectively. As a continuous variable, baseline ANGPTL3/8 was inversely correlated with PH-LPL activity (*r* = −0.23, *P* = 2.2 × 10^−07^) and directly with PH-HL activity (*r* = 0.22, *P* = 1.1 × 10^−06^). Likewise, baseline quintiles of ANGPTL3/8 were negatively related with PH-LPL activity (*P* = 8.2 × 10^−06^ for trend), as PH-LPL activity was the highest in Q1 of ANGPTL3/8 (*P* < 0.03 compared with all other quintiles), whereas the lowest in Q4 and Q5 (*P* < 0.02 compared with Q1 and Q2) even after adjustment for covariates (Table 2). No associations of PH-LPL and PH-HL activities with ANGPTL4/8 were observed in either continuous or quintile models (Table 3).

Quintiles of ANGPTL3/8 and ANGPTL4/8 showed strong relationships with lipids and lipoproteins in the expected directions. For example, levels of both ANGPTL3/8 and ANGPTL4/8 were positively related to total TG (Tables 2 and 3) as well as the TG content of VLDL, LDL, and HDL (supplemental Table S2). ANGPTL3/8 was positively associated with all measured TRLP traits (except small TRLP) and small LDL particles, whereas inversely associated with the concentration of large HDL (particularly the H6 subclass) and HDL size (supplemental Table S2A). ANGPTL4/8 was positively associated with the concentration of total, medium, and small LDL particles and inversely with LDL particle size (supplemental Table S2B). Both complexes were positively related to the concentration of the H2 subspecies (7.8 nm) of HDL (supplemental Table S2).

Baseline quintiles of both ANGPTL complexes showed strong positive associations with BMI and measures of body fat and composition, including fat mass, abdominal visceral fat, and waist circumference (Tables 2 and 3, supplemental Table S3). ANGPTL3/8 and ANGPTL4/8 showed similar relationships with measures of glucose and insulin homeostasis, including positive associations with fasting levels of glucose and insulin and inverse associations with insulin sensitivity index (Tables 2 and 3, supplemental Table S4).

When examined as continuous variables, baseline levels of ANGPTL3/8 and ANGPTL4/8 were significantly (*P* < 9.3 × 10^−04^) correlated with almost all the tested lipid, lipoprotein, body composition, and insulin and glucose traits, except the correlations between ANGPTL4/8 and NMR lipoprotein measures being mostly nominal (*P* < 0.05) (Tables 2 and 3, supplemental Tables S2–S4). Results were comparable to quintile analysis, with the correlations being moderate to weak and generally stronger in ANGPTL3/8 compared with ANGPTL4/8. Nine variables that explained 30% of the variance were retained (of 45 total entered) in a multivariable regression model for baseline ANGPTL3/8. The strongest baseline predictors of ANGPTL3/8 were abdominal fat (partial *R*^2^ = 15.3%) and plasma TG (partial *R*^2^ = 7.7%) followed by very-large TRLP, percent body fat, PH-LPL activity, total cholesterol, self-identified race, TRLP size, and small HDL particle concentration (supplemental Table S5A1). For baseline ANGPTL4/8, four of 40 variables were retained in the regression model, which explained 13.1% of the variance: fasting insulin (partial *R*^2^ = 7.9%), HDL-TG (partial *R*^2^ = 2.7%), insulin sensitivity index (partial *R*^2^ = 1.7%), and body surface area (partial *R*^2^ = 0.8%) (supplemental Table S5B1). Results were similar when only entering traits significantly (*P* < 9.3 × 10^−04^) correlated with ANGPTL3/8 or ANGPTL4/8 at baseline (supplemental Table S5, A2 and B2).

### ANGPTL3/8 and ANGPTL4/8 levels—changes with exercise training

On average, the concentration of both complexes decreased with exercise training, with the decrease in ANGPTL3/8 being statistically significant (Table 1). There was large interindividual variation in the response of the ANGPTL complexes to training (supplemental Figs. S1 and S2). The training response of ANGPTL4/8 differed by sex, with male participants showing a significant decrease, whereas female participants showed no change (Table 1). No differences in exercise response for either ANGPTL complex were found across self-identified race groups or combined race-sex subgroups.

**Fig. 1:**
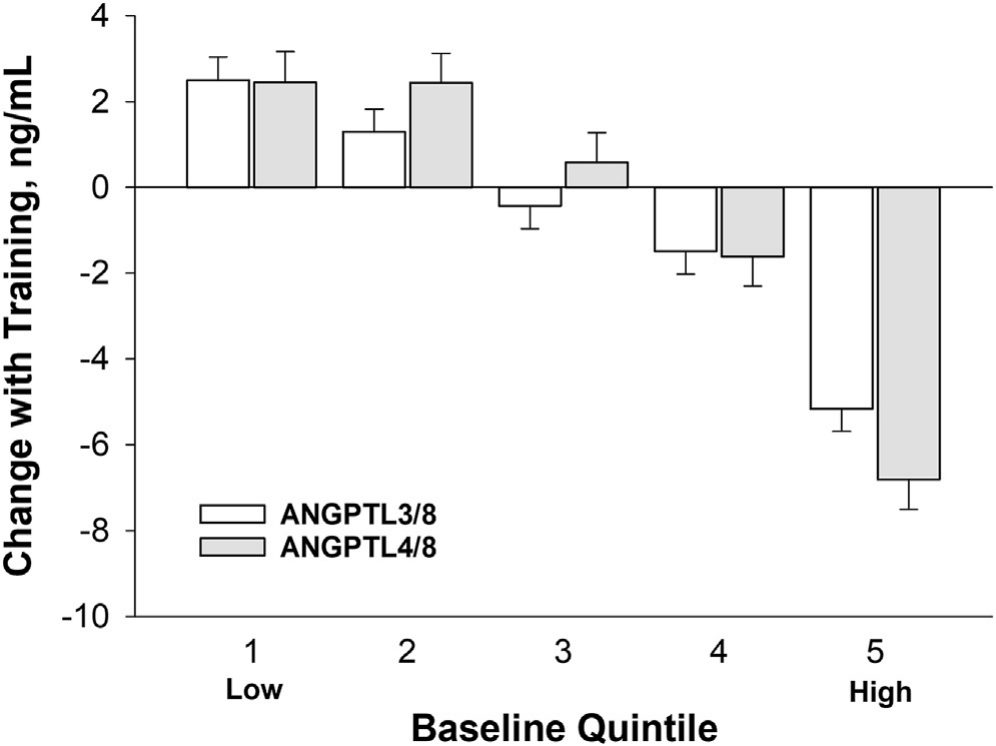
Baseline quintiles of ANGPTL3/8 and ANGPTL4/8 exhibit inverse relationships with their respective exercise-induced changes. Mean values adjusted for age, sex, and race. *P* = 2.4 × 10^−23^ and 1.2 × 10^−23^ for trend across quintiles for ANGPTL3/8 and ANGPTL4/8, respectively. The within-group change from baseline to post-training was significant (*P* < 0.05) in each quintile except Q3 for both ANGPTL3/8 and ANGPTL4/8.

The training responses of ANGPTL3/8 and ANGPTL4/8 were directly correlated with each other (*r* = 0.18, *P* = 3.8 × 10^−06^). Changes in both ANGPTL3/8 (*r* = −0.44, *P* = 1.6 × 10^−32^) and ANGPTL4/8 (*r* = −0.51, *P* = 3.6 × 10^−44^) were also inversely correlated with their respective baseline values. Moreover, baseline quintiles of ANGPTL3/8 and ANGPTL4/8 exhibited inverse linear relationships with their respective exercise-induced changes, with the lowest quintiles showing mean increases with training, whereas the highest quintiles showed progressively larger mean decreases with training (*P* < 2.5 × 10^−23^ for trend) (Fig. 1). The training responses of ANGPTL3/8 and ANGPTL4/8 were significant (*P* < 0.05) within every respective baseline quintile except Q3 (Tables 2 and 3).

### Associations with cardiometabolic traits—changes with exercise training

As a continuous variable, the change in ANGPTL3/8 was significantly, directly correlated with the change in PH-HL activity (*r* = 0.15, *P* = 2.1 × 10^−04^) and nominally, inversely correlated with the change in PH-LPL activity (*r* = −0.13, *P* = 0.002) (Table 2). Conversely, the change in ANGPTL4/8 was nominally, inversely correlated with the change in PH-HL activity (*r* = −0.11, *P* = 0.01) only (Table 3). In general, after accounting for age, sex, race, and baseline trait level, the phenotypic changes with training were similar across baseline quintiles of ANGPTL3/8 and ANGPTL4/8 (Tables 2 and 3). For example, the adjusted mean increase in PH-LPL activity with training was similar across baseline quintiles of ANGPTL3/8 and ANGPTL4/8.

The change in ANGPTL3/8 was significantly but weakly positively correlated with concomitant changes in several lipid traits, including change in total and VLDL cholesterol and TG concentrations, very large and large TRLP concentrations, and TRLP size (Table 2, supplemental Table S2A). The change in ANGPTL4/8 was significantly weakly positively correlated with changes in the concentrations of total and LDL-C and apoB (Table 2, supplemental Table S2B).

After accounting for covariates, the change in ANGPTL3/8 was positively correlated with changes in visceral fat and fat mass (Table 2, supplemental Table S3A), whereas the change in ANGPTL4/8 was not significantly associated with changes in body composition (Table 3, supplemental Table S3B). Change in ANGPTL3/8 was positively correlated with changes in fasting insulin and AIR_g_, whereas change in ANGPTL4/8 was inversely correlated with change in insulin sensitivity index (supplemental Table S4).

In multivariable regression models, eight predictors explained 30% of the variance in change in ANGPTL3/8, including baseline level (partial *R*^2^ = 20.5%) and concomitant changes in very large TRLP (partial *R*^2^ = 5.0%), waist circumference (partial *R*^2^ = 1.3%), PH-HL activity (partial *R*^2^ = 1.1%), AIR_g_, glucose effectiveness, and LDL-C) (supplemental Table S5C). The strongest predictors of change in ANGPTL4/8 were baseline level (partial *R*^2^ = 17.7%) and concomitant changes in waist circumference (partial *R*^2^ = 2.2%), HDL subspecies H2P (partial *R*^2^ = 1.3%), very large TRLP (partial *R*^2^ = 1.1%), and LDL-C (partial *R*^2^ = 0.7%) (supplemental Table S5D).

## Discussion

Our study characterized ANGPTL3/8 and ANGPTL4/8 levels before and after exercise training and compared these levels with lipolytic activities, and lipid, lipoprotein, and other cardiometabolic traits in the HERITAGE Family Study. Novel findings of our study include the inverse and direct association of ANGPTL3/8 with PH-LPL and PH-HL activities, respectively, and the association of both ANGPTL complexes with comprehensive measures of lipids and lipoproteins and their subclasses, body composition and abdominal fat, and glucose and insulin homeostasis from IVGTT. Our findings are similar to a report by our group, which showed that both ANGPTL3/8 and ANGPTL4/8 were inversely correlated with HDL-C and directly correlated with TG, fasting glucose, fasting insulin, waist to hip ratio, and BMI in 352 healthy adults, whereas only ANGPTL3/8 was correlated with LDL-C (15). Chen *et al.* (20) also found that both ANGPTL complexes were positively correlated with TG, whereas ANGPTL3/8 was correlated with total cholesterol and non-HDL-C and ANGPTL4/8 inversely correlated with HDL-C in type 2 diabetics.

Similar to previous reports (15, 20), the positive correlation of circulating ANGPTL4/8 with plasma TG is not fully understood. A recent report by our group showed that LPL-bound ANGPTL4/8 catalyzes the generation of plasmin in the adipose tissue, which can then cleave ANGPTL4/8 (19). Although ANGPTL4/8 in the adipose tissue protects LPL from circulating inhibitors such as ANGPTL3/8 (15, 42), the ANGPTL4/8-plasmin model suggests that while plasmin cleavage of ANGPTL4/8 bound to LPL may cause an initial increase in LPL activity, it could also leave LPL exposed to circulating inhibitors that might decrease LPL activity and thus ultimately result in increased circulating TG. However, the work from Oldoni *et al.* (42) suggests that circulating ANGPTL4/8 may come from the liver. ANGPTL4/8 secreted by the liver directly into the circulation would likely not be cleaved by plasmin. We do not currently know what stimuli cause increased ANGPTL4/8 secretion from the liver, and there are both consistencies and discrepancies between existing biochemical and in vivo studies. Thus, this is an area in need of further investigation.

Interestingly, we found that ANGPTL3/8 was positively correlated with PH-HL activity. As we are the first to report this relationship, the mechanisms are unknown, but the most likely explanation is that higher insulin levels cause increased HL activity and ANGPTL3/8 secretion from the liver. But ANGPTL3/8 does not inhibit HL (43, 44), thus levels of ANGPTL3/8 are directly correlated with HL activity.

A major finding of our study was that on average regular exercise significantly decreased ANGPTL3/8 levels by 6%, whereas ANGPTL4/8 did not significantly change (4% decrease). However, when examined across baseline quintiles, the fourth and fifth quintiles of both baseline ANGPTL3/8 and ANGPTL4/8 exhibited significant stepwise decreases with exercise training. To our knowledge, this represents the first report on the effects of regular exercise on the ANGPTL3/8 and ANGPTL4/8 complexes. Moreover, exercise-induced changes in several lipid traits were related to concomitant changes in ANGPTL3/8 and ANGPTL4/8. The change in ANGPTL3/8 was directly associated with changes in the concentration of TG and TRLP subclasses. We hypothesize that the reduction in ANGPTL3/8 complexes with exercise training may allow for greater LPL activation in muscle, which in turn contributes to reduced circulating TG levels. However, our reported associations are observational in nature, and any putative mechanisms need to be experimentally validated in model systems. Our findings of exercise-induced decreases in ANGPTL3/8 levels and concomitant improvements in the lipid and lipoprotein profile are encouraging from a clinical perspective, given the results from a recent phase 1 randomized, double-blind, placebo-controlled trial of a monoclonal antibody against the ANGPTL3/8 complex (LY345766), which showed dose-dependent decreases in free ANGPTL3/8 levels that were accompanied by dose-dependent decreases in TG, LDL-C, and non-HDL-C and increases in HDL-C up to 2 weeks after injection (22). Similarly, an anti-ANGPTL3/8 antibody was shown to block ANGPTL3/8-mediated LPL inhibition in vitro and potently lower TG in vivo (21).

A limited number of studies have examined the effects of chronic exercise or habitual physical activity on circulating levels of individual ANGPTL3, 4, and 8 proteins, with more assessing the effects of acute bouts of exercise. Previous studies have identified ANGPTL4 as an “exerkine” (45), with small studies generally showing that ANGPTL4 expression increases in muscle, adipose, and hepatic tissues with acute exercise (8, 29, 30, 46), whereas the effects of exercise training on ANGPTL4 levels are mixed (46, 47). In general, exercise and lifestyle interventions appear to decrease ANGPTL8 levels in obese adults (48, 49, 50), with two studies showing similar decreases between the diet-only and diet plus exercise groups (49, 50). To our knowledge, the impact of acute exercise or chronic exercise on ANGPTL3 expression has not been studied. A cross-sectional study of 22 physically active students and 28 age-matched sedentary students found no difference in serum ANGPTL3 between the groups (51).

It is difficult to directly compare the results from small studies on the effects of mostly acute exercise or lifestyle interventions on individual ANGPTL proteins to the current large study on exercise training and the ANGPTL3/8 and ANGPTL4/8 complexes. One limitation is the different physiological activities between the circulating individual proteins compared with their complexes, as ANGPTL3 alone has low LPL-inhibiting activity, whereas ANGPTL4 by itself strongly inhibits LPL, and the converse is true when each is complexed with ANGPTL8. Moreover, fasting and feeding conditions are more likely to have transient effects on ANGPTL3/4/8 levels and be tissue specific rather than reflect chronic serum levels per se. Thus, it is not clear how the acute effects of exercise compare to the chronic effects of exercise training on circulating levels of ANGPTL3/8 and ANGPTL4/8 complexes. Also, lifestyle interventions result in modest changes in physical activity resulting from divergent forms of physical activity and/or exercise, which may not be comparable to controlled prescribed doses of exercise training.

Our study is the first to show that higher levels of ANGPTL3/8 and ANGPTL4/8 are associated with lower insulin sensitivity and that exercise-induced changes in both complexes were inversely associated with concomitant changes in insulin homeostasis. Interestingly, fasting insulin and insulin sensitivity index were two of the four significant predictors of baseline ANGPTL4/8 levels. Our group previously showed an important role of insulin stimulation on the secretion of each complex, with insulin increasing ANGPTL3/8 secretion from hepatocytes and ANGPTL4/8 secretion from adipocytes (15). The finding of exercise-induced reductions in circulating ANGPTL3/8 and ANGPTL4/8 levels leading to concomitant improvements of markers of insulin homeostasis may appear counterintuitive, as it might be expected that reduced ANGPTL complex levels would increase TG uptake into skeletal muscle, which could potentially increase insulin resistance in muscle. Although the exact mechanisms underlying this interrelationship are unknown, a recent collaboration involving our group showed that there is decreased LPL in capillaries of oxidative tissue in ApoA5 knockout mice because of unrestrained ANGPTL3/8 activity (52). However, when ANGPTL3/8 was blocked, intracapillary LPL levels returned to normal but did not exceed normal levels. Thus, “lower” amounts of active ANGPTL3/8 should not result in excessive uptake of TG into oxidative tissue.

We observed large heterogeneity in the responses of ANGPTL3/8 and ANGPTL4/8 to regular exercise, with some individuals showing large decreases and others large increases. Some of this heterogeneity appears related to baseline levels, as our multivariable regression models showed that baseline level was the strongest predictor of exercise-induced change in both ANGPTL3/8 and ANGPTL4/8 and explained 17–19% of the variance in these changes. As expected, changes in many lipid traits were associated with concomitant changes in ANGPTL3/8 and ANGPTL4/8. The change in ANGPTL3/8 was directly associated with changes in the concentration of TG and TRLPs, whereas change in AGPTL4/8 was correlated with changes in the TG content of HDL and LDL particles. These findings may represent mechanistic differences between ANGPTL3/8 and ANGPTL4/8. Since the binding of ANGPTL8 to ANGPTL4 limits the inhibitory capacity of ANGPTL4 on LPL, it is likely that the generally stronger associations observed with ANGPTL3/8 compared with ANGPTL4/8 reflect this weakened antagonism. Moreover, change in ANGPTL3/8 was associate d with changes in total fat and visceral fat. We found waist circumference was one of the strongest correlates of both baseline levels and the change in ANGPTL4/8, which corresponds with in vivo experiments that showed ANGPTL4/8 is secreted by adipocytes and remains mostly localized in adipose tissue, and circulating ANGPTL4/8 levels may reflect adipose tissue ANGPTL4/8 levels (15).

There are several strengths to our study, including a large sample size of racially diverse adults, a standardized and supervised exercise program with excellent adherence, and the dedicated assays being performed in a single laboratory with high reproducibility (15, 20, 21). Moreover, we included measures of PH lipase activities, lipoprotein subclasses, IVGTT, and objective measures of body composition. However, our study is not without limitations that may limit the generalizability of the observations. Importantly, the HERITAGE study did not include a control group, and the participants were generally healthy. Thus, we cannot rule out the possibility of the regression toward the mean phenomenon; however, the changes in the traditional lipid traits and lipid subclasses align with those observed in numerous other exercise training studies supporting likely “real” training effects on lipid and lipoprotein metabolism. Although not a major concern given the strong correlation between blood matrices (53, 54), it is worth noting that the ANGPTL complex assays were performed on serum, whereas the included lipid and lipoprotein traits were measured in plasma, potentially attenuating the associations observed.

## Conclusions

Our findings provide insights into the potentially differential biological mechanisms underlying ANGPTL3/8 and ANGPTL4/8 complexes in the untrained and exercise trained states. Overall, we found that regular exercise reduces the levels of the ANGPTL3/8 and ANGPTL4/8 complexes in individuals who started with the highest levels and that their responses to regular exercise relate to the concomitant exercise-induced changes in lipases, lipids and lipoproteins, body composition, and insulin and glucose metabolism. Thus, these findings reveal the ANGPTL3-4-8 model as a potential novel molecular mechanism contributing to otherwise well-studied adaptations in lipid metabolism in response to exercise training. Moreover, these findings reflect the potential beneficial effects of regular exercise on the ANGPTL complexes, although the full scope of these effects will require further study including testing different doses and types of exercise in healthy and diseased populations.

## Data availability

The data that support the findings of this study are available from the corresponding author, Mark A. Sarzynski (Department of Exercise Science, University of South Carolina, sarz@mailbox.sc.edu), upon reasonable request.

## Supplemental data

This article contains supplemental data.

## Conflict of interest

Y. Q. C. and R. J. K. are Eli Lilly and Company employees and own Lilly stock. M. A. S. reports that financial support was provided by the National Institutes of Health. G. S. reports that financial support was provided by Eli Lilly and Company. J. S. M. M. reports that financial support was provided by the National Institutes of Health. R. E. G. reports that financial support was provided by the National Institutes of Health. R. J. K. and Y. Q. C. report a relationship with Eli Lilly and Company that includes employment and equity or stocks. All other authors declare that they have no conflicts of interest with the contents of this article.

## References

[1] Shimizugawa T., Ono M., Shimamura M., Yoshida K., Ando Y., Koishi R. (2002). ANGPTL3 decreases very low density lipoprotein triglyceride clearance by inhibition of lipoprotein lipase. J. Biol. Chem..

[2] Quagliarini F., Wang Y., Kozlitina J., Grishin N.V., Hyde R., Boerwinkle E. (2012). Atypical angiopoietin-like protein that regulates ANGPTL3. Proc. Natl. Acad. Sci. U. S. A..

[3] Yoshida K., Shimizugawa T., Ono M., Furukawa H. (2002). Angiopoietin-like protein 4 is a potent hyperlipidemia-inducing factor in mice and inhibitor of lipoprotein lipase. J. Lipid Res..

[4] Koster A., Chao Y.B., Mosior M., Ford A., Gonzalez-DeWhitt P.A., Hale J.E. (2005). Transgenic angiopoietin-like (angptl)4 overexpression and targeted disruption of angptl4 and angptl3: regulation of triglyceride metabolism. Endocrinology.

[5] Zhang R. (2012). Lipasin, a novel nutritionally-regulated liver-enriched factor that regulates serum triglyceride levels. Biochem. Biophys. Res. Commun..

[6] Ploug M. (2022). ANGPTL4: a new mode in the regulation of intravascular lipolysis. Curr. Opin. Lipidol..

[7] Kersten S., Mandard S., Tan N.S., Escher P., Metzger D., Chambon P. (2000). Characterization of the fasting-induced adipose factor FIAF, a novel peroxisome proliferator-activated receptor target gene. J. Biol. Chem..

[8] Kersten S., Lichtenstein L., Steenbergen E., Mudde K., Hendriks H.F., Hesselink M.K. (2009). Caloric restriction and exercise increase plasma ANGPTL4 levels in humans via elevated free fatty acids. Arterioscler. Thromb. Vasc. Biol..

[9] Uhlen M., Fagerberg L., Hallstrom B.M., Lindskog C., Oksvold P., Mardinoglu A. (2015). Proteomics. Tissue-based map of the human proteome. Science.

[10] Wang Y., McNutt M.C., Banfi S., Levin M.G., Holland W.L., Gusarova V. (2015). Hepatic ANGPTL3 regulates adipose tissue energy homeostasis. Proc. Natl. Acad. Sci. U. S. A..

[11] Ginsberg H.N., Goldberg I.J. (2022). Broadening the scope of dyslipidemia therapy by targeting APOC3 and ANGPTL3 (Angiopoietin-Like protein 3). Arterioscler. Thromb. Vasc. Biol..

[12] Ruscica M., Zimetti F., Adorni M.P., Sirtori C.R., Lupo M.G., Ferri N. (2020). Pharmacological aspects of ANGPTL3 and ANGPTL4 inhibitors: new therapeutic approaches for the treatment of atherogenic dyslipidemia. Pharmacol. Res..

[13] Chi X., Britt E.C., Shows H.W., Hjelmaas A.J., Shetty S.K., Cushing E.M. (2017). ANGPTL8 promotes the ability of ANGPTL3 to bind and inhibit lipoprotein lipase. Mol. Metab..

[14] Kovrov O., Kristensen K.K., Larsson E., Ploug M., Olivecrona G. (2019). On the mechanism of angiopoietin-like protein 8 for control of lipoprotein lipase activity. J. Lipid Res..

[15] Chen Y.Q., Pottanat T.G., Siegel R.W., Ehsani M., Qian Y.W., Zhen E.Y. (2020). Angiopoietin-like protein 8 differentially regulates ANGPTL3 and ANGPTL4 during postprandial partitioning of fatty acids. J. Lipid Res..

[16] Zhang R. (2016). The ANGPTL3-4-8 model, a molecular mechanism for triglyceride trafficking. Open Biol..

[17] Zhang R., Zhang K. (2022). An updated ANGPTL3-4-8 model as a mechanism of triglyceride partitioning between fat and oxidative tissues. Prog. Lipid Res..

[18] Jin N., Matter W.F., Michael L.F., Qian Y., Gheyi T., Cano L. (2021). The angiopoietin-like protein 3 and 8 complex Interacts with lipoprotein lipase and Induces LPL cleavage. ACS Chem. Biol..

[19] Zhen E.Y., Chen Y.Q., Russell A.M., Ehsani M., Siegel R.W., Qian Y. (2023). Angiopoietin-like protein 4/8 complex-mediated plasmin generation leads to cleavage of the complex and restoration of LPL activity. Proc. Natl. Acad. Sci. U. S. A..

[20] Chen Y.Q., Pottanat T.G., Siegel R.W., Ehsani M., Qian Y.W., Konrad R.J. (2021). Angiopoietin-like protein 4 (ANGPTL4) is an inhibitor of endothelial lipase (EL) while the ANGPTL4/8 complex has reduced EL-inhibitory activity. Heliyon.

[21] Balasubramaniam D., Schroeder O., Russell A.M., Fitchett J.R., Austin A.K., Beyer T.P. (2022). An anti-ANGPTL3/8 antibody decreases circulating triglycerides by binding to a LPL-inhibitory leucine zipper-like motif. J. Lipid Res..

[22] Gaudet D., Gonciarz M., Shen X., Mullins G., Leohr J.K., Benichou O. (2022). A first-in-human single ascending dose study of a monoclonal antibody against the ANGPTL3/8 complex in subjects with mixed hyperlipidemia. Atherosclerosis.

[23] National Cholesterol Education Program (2002). Third report of the National cholesterol Education program (NCEP) expert panel on detection, evaluation, and treatment of high blood cholesterol in adults (adult treatment Panel III) final report. Circulation.

[24] Durstine J.L., Grandjean P.W., Davis P.G., Ferguson M.A., Alderson N.L., DuBose K.D. (2001). Blood lipid and lipoprotein adaptations to exercise: a quantitative analysis. Sports Med..

[25] Chapman M.J., Ginsberg H.N., Amarenco P., Andreotti F., Boren J., Catapano A.L. (2011). Triglyceride-rich lipoproteins and high-density lipoprotein cholesterol in patients at high risk of cardiovascular disease: evidence and guidance for management. Eur. Heart J..

[26] Bergeron J., Couillard C., Despres J.P., Gagnon J., Leon A.S., Rao D.C. (2001). Race differences in the response of postheparin plasma lipoprotein lipase and hepatic lipase activities to endurance exercise training in men: results from the HERITAGE family study. Atherosclerosis.

[27] Ferguson M.A., Alderson N.L., Trost S.G., Essig D.A., Burke J.R., Durstine J.L. (1998). Effects of four different single exercise sessions on lipids, lipoproteins, and lipoprotein lipase. J. Appl. Physiol. (1985).

[28] Kantor M.A., Cullinane E.M., Sady S.P., Herbert P.N., Thompson P.D. (1987). Exercise acutely increases high density lipoprotein-cholesterol and lipoprotein lipase activity in trained and untrained men. Metabolism.

[29] Catoire M., Alex S., Paraskevopulos N., Mattijssen F., Evers-van Gogh I., Schaart G. (2014). Fatty acid-inducible ANGPTL4 governs lipid metabolic response to exercise. Proc. Natl. Acad. Sci. U. S. A..

[30] Ingerslev B., Hansen J.S., Hoffmann C., Clemmesen J.O., Secher N.H., Scheler M. (2017). Angiopoietin-like protein 4 is an exercise-induced hepatokine in humans, regulated by glucagon and cAMP. Mol. Metab..

[31] Slade S.C., Dionne C.E., Underwood M., Buchbinder R., Beck B., Bennell K. (2016). Consensus on exercise reporting Template (CERT): modified delphi study. Phys. Ther..

[32] Bouchard C., Leon A.S., Rao D.C., Skinner J.S., Wilmore J.H., Gagnon J. (1995). The HERITAGE family study. Aims, design, and measurement protocol. Med. Sci. Sports Exerc..

[33] Sarzynski M.A., Rice T.K., Despres J.P., Perusse L., Tremblay A., Stanforth P.R. (2022). The HERITAGE family study: a review of the effects of exercise training on cardiometabolic health, with insights into molecular transducers. Med. Sci. Sports Exerc..

[34] Burstein M., Samaille J. (1960). On a rapid determination of the cholesterol bound to the serum alpha- and beta-lipoproteins. Clin. Chim. Acta.

[35] St-Amand J., Moorjanit S., Lupien P.J., Prud'homme D., Despres J.P. (1996). The relation of plasma triglyceride, apolipoprotein B, and high-density lipoprotein cholesterol to postheparin lipoprotein lipase activity is dependent on apolipoprotien E polymorphism. Metabolism.

[36] Gagnon J., Province M.A., Bouchard C., Leon A.S., Skinner J.S., Wilmore J.H. (1996). The HERITAGE family study: quality assurance and quality control. Ann. Epidemiol..

[37] Despres J.P., Gagnon J., Bergeron J., Couillard C., Leon A.S., Rao D.C. (1999). Plasma post-heparin lipase activities in the HERITAGE Family Study: the reproducibility, gender differences, and associations with lipoprotein levels. HEalth, RIsk factors, exercise Training and GEnetics. Clin. Biochem..

[38] Jeyarajah E.J., Cromwell W.C., Otvos J.D. (2006). Lipoprotein particle analysis by nuclear magnetic resonance spectroscopy. Clin. Lab. Med..

[39] Wilmore J.H., Stanforth P.R., Domenick M.A., Gagnon J., Daw E.W., Leon A.S. (1997). Reproducibility of anthropometric and body composition measurements: the HERITAGE family study. Int. J. Obes. Relat. Metab. Disord..

[40] Wilmore J.H., Despres J.P., Stanforth P.R., Mandel S., Rice T., Gagnon J. (1999). Alterations in body weight and composition consequent to 20 wk of endurance training: the HERITAGE Family Study. Am. J. Clin. Nutr..

[41] Boston R.C., Stefanovski D., Moate P.J., Sumner A.E., Watanabe R.M., Bergman R.N. (2003). MINMOD Millennium: a computer program to calculate glucose effectiveness and insulin sensitivity from the frequently sampled intravenous glucose tolerance test. Diabetes Technol. Ther..

[42] Oldoni F., Cheng H., Banfi S., Gusarova V., Cohen J.C., Hobbs H.H. (2020). ANGPTL8 has both endocrine and autocrine effects on substrate utilization. JCI Insight.

[43] Wang Y., Quagliarini F., Gusarova V., Gromada J., Valenzuela D.M., Cohen J.C. (2013). Mice lacking ANGPTL8 (Betatrophin) manifest disrupted triglyceride metabolism without impaired glucose homeostasis. Proc. Natl. Acad. Sci. U. S. A..

[44] Baynes C., Henderson A.D., Anyaoku V., Richmond W., Hughes C.L., Johnston D.G. (1991). The role of insulin insensitivity and hepatic lipase in the dyslipidaemia of type 2 diabetes. Diabet. Med..

[45] Chow L.S., Gerszten R.E., Taylor J.M., Pedersen B.K., van Praag H., Trappe S. (2022). Exerkines in health, resilience and disease. Nat. Rev. Endocrinol..

[46] Norheim F., Hjorth M., Langleite T.M., Lee S., Holen T., Bindesboll C. (2014). Regulation of angiopoietin-like protein 4 production during and after exercise. Physiol. Rep..

[47] Cullberg K.B., Christiansen T., Paulsen S.K., Bruun J.M., Pedersen S.B., Richelsen B. (2013). Effect of weight loss and exercise on angiogenic factors in the circulation and in adipose tissue in obese subjects. Obesity (Silver Spring).

[48] Abu-Farha M., Sriraman D., Cherian P., AlKhairi I., Elkum N., Behbehani K. (2016). Circulating ANGPTL8/Betatrophin is increased in obesity and reduced after exercise training. PLoS One.

[49] Fu C.P., Oczypok E.E., Ali H., DeLany J.P., Reeves V.L., Chang R.F. (2022). Effect of physical activity in a weight loss program on circulating total ANGPTL8 concentrations in northern Americans with obesity: a prospective randomized controlled trial. Nutr. Metab. Cardiovasc. Dis..

[50] Hu H., Yuan G., Wang X., Sun J., Gao Z., Zhou T. (2019). Effects of a diet with or without physical activity on angiopoietin-like protein 8 concentrations in overweight/obese patients with newly diagnosed type 2 diabetes: a randomized controlled trial. Endocr. J..

[51] Smol E., Klapcinska B., Kempa K., Fredyk A., Malecki A. (2015). Effects of regular Recreational exercise training on serum ANGPTL3-like protein and lipid profile in Young healthy adults. J. Hum. Kinet..

[52] Yang Y., Beigneux A.P., Song W., Nguyen L.P., Jung H., Tu Y. (2023). Hypertriglyceridemia in Apoa5-/- mice results from reduced amounts of lipoprotein lipase in the capillary lumen. J. Clin. Invest.

[53] (1977). Cholesterol and triglyceride concentrations in serum/plasma pairs. Clin. Chem..

[54] Beheshti I., Wessels L.M., Eckfeldt J.H. (1994). EDTA-plasma vs serum differences in cholesterol, high-density-lipoprotein cholesterol, and triglyceride as measured by several methods. Clin. Chem..

